# Increasing thermal stress for tropical coral reefs: 1871–2017

**DOI:** 10.1038/s41598-018-24530-9

**Published:** 2018-04-17

**Authors:** J. M. Lough, K. D. Anderson, T. P. Hughes

**Affiliations:** 1 Australian Institute of Marine Science, PMB3, Townsville MC, QLD 4810 Australia; 2 ARC Centre of Excellence for Coral Reef Studies, James Cook University, Townsville, QLD 4811 Australia

## Abstract

Tropical corals live close to their upper thermal limit making them vulnerable to unusually warm summer sea temperatures. The resulting thermal stress can lead to breakdown of the coral-algal symbiosis, essential for the functioning of reefs, and cause coral bleaching. Mass coral bleaching is a modern phenomenon associated with increases in reef temperatures due to recent global warming. Widespread bleaching has typically occurred during El Niño events. We examine the historical level of stress for 100 coral reef locations with robust bleaching histories. The level of thermal stress (based on a degree heating month index, DHMI) at these locations during the 2015–2016 El Niño was unprecedented over the period 1871–2017 and exceeded that of the strong 1997–1998 El Niño. The DHMI was also 5 times the level of thermal stress associated with the ‘pre-industrial’, 1877–1878, El Niño. Coral reefs have, therefore, already shown their vulnerability to the modest (~0.92 °C) global warming that has occurred to date. Estimates of future levels of thermal stress suggest that even the optimistic 1.5 °C Paris Agreement target is insufficient to prevent more frequent mass bleaching events for the world’s reefs. Effectively, reefs of the future will not be the same as those of the past.

## Introduction

As the global climate system warms so do the tropical oceans and that is not good news for coral reefs. Tropical corals live close to their upper thermal limit and relatively small excursions of sea surface temperature (SST) above the average summer maximum can lead to the breakdown of the coral-algal symbiosis^1^. This mutually beneficial relationship between the coral animal and photosynthetic microscopic plants (zooxathellae, *Symbiodinium*) is the foundation of modern coral reef ecosystems. The coral host obtains photosynthetic products from the algae and this extra source of cheap energy allows the coral to calcify faster than the natural forces of physical and biological erosion^2^. The resulting sustained building of calcium carbonate skeletons is the backbone of the reef, providing habitat for many thousands of reef-associated organisms that together make up a tropical coral reef ecosystem^3^. These ecosystems are a vast storehouse of biodiversity and are estimated to contain about 25% of all marine species^4^ yet the combined area of the world’s shallow water reefs is only about half the size of France. The loss of the algae, whose photosynthetic pigments provide corals with their colour, results in the coral tissue becoming translucent and the white skeleton is then visible – hence the term ‘coral bleaching’^1^. Once the thermal stress is removed, there are various outcomes for the coral – some die, some partially die, some recover and some may not be affected at all^5^. For surviving corals, reproduction rates may be reduced^6^, growth rates slowed^7^, and the prevalence of coral diseases increased^8^. Dead coral are often then overgrown by macroalgae (seaweeds), but eventually erode and breakdown and there can be both immediate and long-term consequences for reef-associated organisms (e.g. decline in fish abundance and change in community make-up^9^). Mass coral bleaching events, where large tracts of reefs are affected, appears to be a relatively modern phenomenon first reported in the early 1980s^10^. The major trauma of such events, and their apparent and projected increase in frequency, has significant consequences for the maintenance of present-day reef ecosystems and is recognized as a potentially profound consequence of a rapidly changing global climate^1,2,11–13^.

The link between mass coral bleaching events and unusually warm summer SST is unequivocal and now forms the basis of a global thermal stress monitoring system^14,15^ (http://coralreefwatch.noaa.gov/satellite/index.php). That mass bleaching events had a global signature and apparent links with El Niño events^16^ was dramatically confirmed during the 1997–1998 El Niño when bleaching affected nearly every coral reef region^17,18^. The aggregate level of thermal stress across 47 affected reefs during the 1997–1998 El Niño was unprecedented over the period 1903–1999^19^. Mass coral bleaching events have continued since then, for example, ~50% of Australia’s Great Barrier Reef was affected in 2002^20^, ~80% of Caribbean reefs in 2005^21^ and many globally-distributed sites in 2010^22,23^. In mid-2014 bleaching was reported in the western northern tropical Pacific and progressed through many of the world’s reef systems through 2017, tracking the local seasonal maximum SST at the different reef locations^24,25^. The major El Niño event of 2015–2016^26^ resulted in significant warming of large areas of the tropical oceans and continued bleaching of substantial areas of reef, including Australia’s Great Barrier Reef in early 2016^27^.

Given this recent extensive coral bleaching associated with the major 2015–2016 El Niño event, which is occurring against a backdrop of global warming, this study aims to (1) document how warming of coral reef SST compares to global average warming, (2) determine the level of thermal stress experienced at 100 reef locations with robust bleaching histories^28^ in 2015–2016, (3) place the recent thermal stress in an historical context, e.g. how does the level of thermal stress compare with the very strong ‘pre-industrial’ El Niño of 1877–1878^29,30^? and (4) what estimated level of thermal stress might be expected at these selected reef sites, given current warming rates, if global warming is limited to 1.5 °C, 2.0 °C as per the Paris Agreement^31^ or 3 °C, the trajectory based on the Nationally Determined Contributions of individual nations under the UNFCC^32^ (NDCs)?

## Results

**Figure 1:**
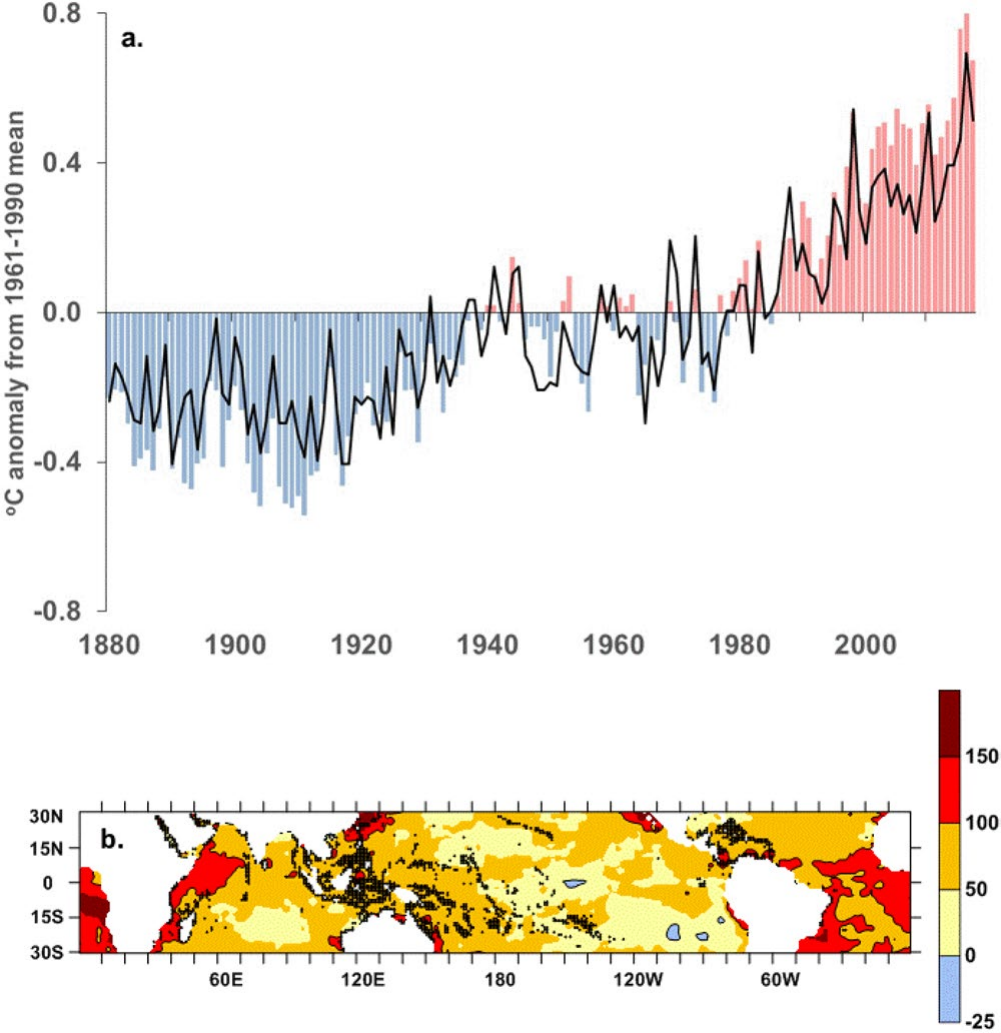
(**a**) Annual average global land and sea temperature (HadCRUT4, red and blue bars) and average annual tropical coral reef sea surface temperature (HadISST1, black line) as anomalies from 1961–1990 average, 1880–2017, and (**b**) annual tropical sea surface temperature warming as percentage of global land and sea temperature warming, 1880–2017. Black symbols indicate 1-degree latitude by longitude boxes containing 1,670 tropical coral reefs.

Over the period 1880–2012, the latest IPCC AR5 assessment^33^ reported warming of global land and sea temperatures by 0.85 °C. Updating this record through 2017, shows an overall warming of 0.92 °C and 2016 was the warmest year on record, exceeding that of 2015^34^. Warming of average annual SST at 1,670 global tropical coral reef sites has largely tracked that of global average temperatures (Fig. 1a) though at a slightly lower rate of 0.65 °C, over the period 1880–2017 (71% of the global average). There is also considerable spatial variability in how the tropical oceans have warmed relative to global average temperatures (Fig. 1b). Warming of the tropical oceans has been substantially less than the global average rate throughout much of the central and eastern tropical Pacific with some small areas (<1%) actually cooling slightly since 1880. 5% of the tropical oceans have warmed <25% of the global average; 55% have warmed 25–75% of the global average; 24% have warmed 75–100% of the global average; with some regions (16% of the tropical oceans) such as the near-equatorial western Indian Ocean warming more than the global average.

**Figure 2:**
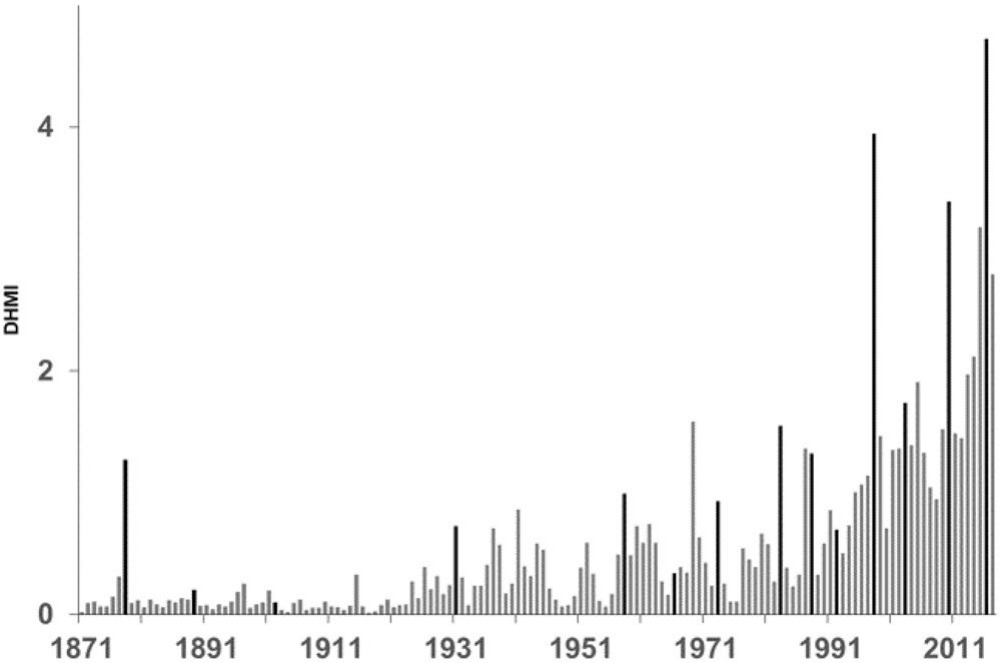
Average annual degree heating month index (DHMI) for 100 coral reef locations, 1871–2017 (grey bars). Black bars mark years with Niño 3.4 index ≥1.0 °C.

The average degree heating month index (DHMI; a measure of both the magnitude and duration of warm season SST anomalies) of 4.7 for 100 globally-distributed reef locations^28^ in 2016 was the highest on record between 1871 and 2017 (Fig. 2; Table 1). The second most extreme year was 1998 with a DHMI of 3.9. In 2016 the DHMI ranged from no stress (0.0) at Mauritius and the Cook Islands to a maximum of 14.0 for the Chagos Archipelago. For 20% of locations the thermal stress index was ≤2.0; 53% with thermal stress of 2.0–6.0 and 27% with thermal stress ≥8.0.

**Table 1:** Observed average DHMI for five El Niño events and estimated values if global warming is constrained to 1.5 °C, 2.0 °C and 3.0 °C above pre-industrial values, for 100 reef locations and four regions (number of locations in each region in parenthesis). DHMI values are for the second year of each event (e.g. 1998 for 1997–1998 event) and values in parentheses are summed index across two years of each El Niño event (e.g. 1997 and 1998).

	All (100)	Indian Ocean/Middle East (28)	SE Asia (14)	Pacific Ocean (36)	Caribbean/Atlantic (22)
1877–1878	1.3 (1.6)	1.9 (2.4)	1.4 (1.5)	0.4 (0.6)	1.9 (2.3)
1982–1983	1.6 (1.8)	2.3 (2.4)	2.0 (2.2)	1.2 (1.7)	0.8 (0.9)
1997–1998	3.9 (5.1)	5.3 (5.9)	5.0 (6.3)	2.8 (4.0)	3.4 (5.0)
2009–2010	3.4 (4.9)	3.6 (4.9)	4.2 (5.0)	1.7 (3.1)	5.3 (7.9)
2015–2016	4.7 (7.9)	5.4 (7.9)	6.5 (9.6)	3.1 (5.5)	5.5 (10.7)
+1.5 °C	8.5	7.8	13.4	5.9	10.4
+2.0 °C	14.6	14.1	24.9	10.2	15.8
+3.0 °C	30.1	30.7	50.7	21.4	30.8

**Figure 3:**
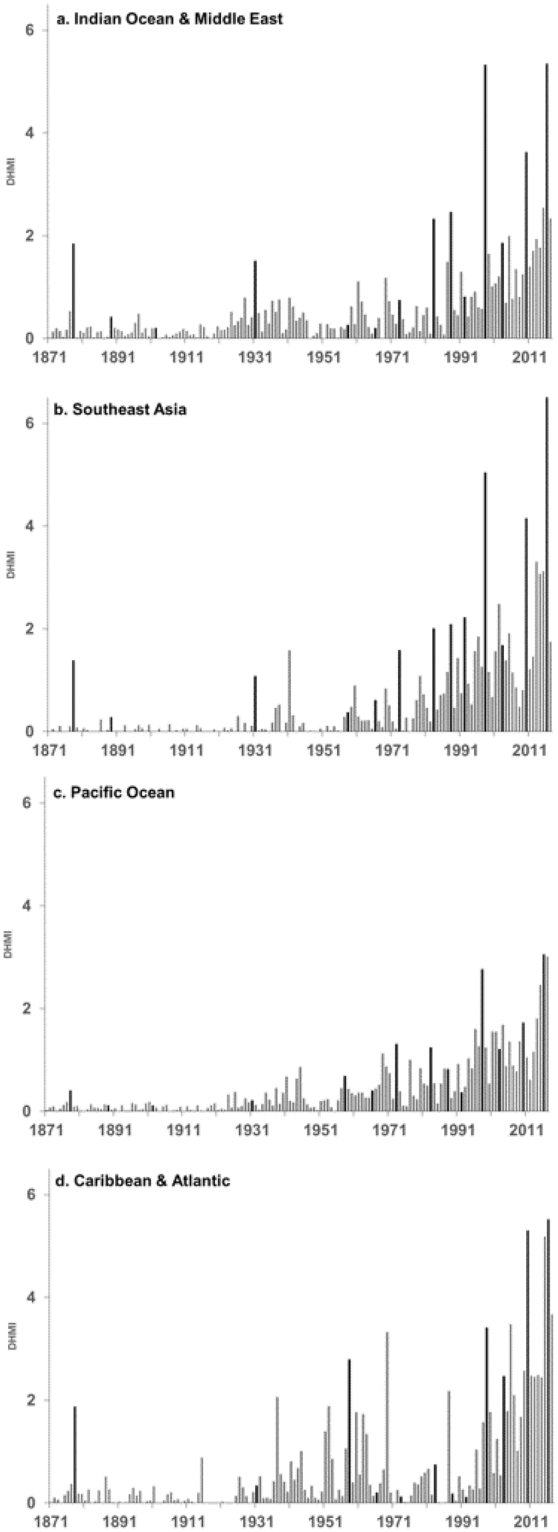
Average annual degree heating month index (DHMI), 1871–2017, (grey bars) for (**a**) Indian Ocean and Middle East, (**b**) Southeast Asia, (**c**) Pacific Ocean and (**d**) Caribbean and Atlantic. Black bars mark years with Niño 3.4 index ≥1.0 °C.

The most extreme years of thermal stress at the selected sites have also, almost invariably, been associated with major El Niño events (highlighted in Fig. 2). The very strong ‘pre-industrial’ El Niño of the late 19^th^ century was associated with a DHMI in 1878 of 1.3. The level of thermal stress associated with El Niño’s and major documented coral bleaching has escalated since the late 19^th^ century. Relative to 1878 the level of thermal stress was 1.2 times greater in 1983, 3 times greater in 1998, 2.6 times greater in 2010 and 3.6 times greater in 2016. As El Niño’s usually evolve over two years (as well as their impacts on reefs) if we combine the total thermal stress for these events then for 1982–1983 the level was 1.1 times, 1997–1998 3.2 times, 2009–2010 3.1 times and 2015–2016 4.9 times the thermal stress experienced in 1877–1878 of 1.6 DHMI. There were also regional differences in the temporal evolution of thermal stress, 1871–2017, across the 100 reef locations (Fig. 3; Table S1). The DHMI values were generally lower in the Pacific Ocean (Fig. 3c) and there was an earlier emergence of higher levels of thermal stress in the Caribbean and Atlantic^19^ (Fig. 3d). All series were significantly correlated (at the 5% level) with the Niño 3.4 index, 1871–2017: r = 0.43 for all 100 locations, 0.48 for the Indian Ocean and Middle East, 0.48 for Southeast Asia, 0.34 for the Pacific Ocean and 0.32 for the Caribbean and Atlantic. Also noteworthy, is the DHMI for 2017 which, although less than in 2016, is still high relative to values in the 1980s, when global mass coral bleaching events first started to emerge (Figs 2 and 3).

**Figure 4:**
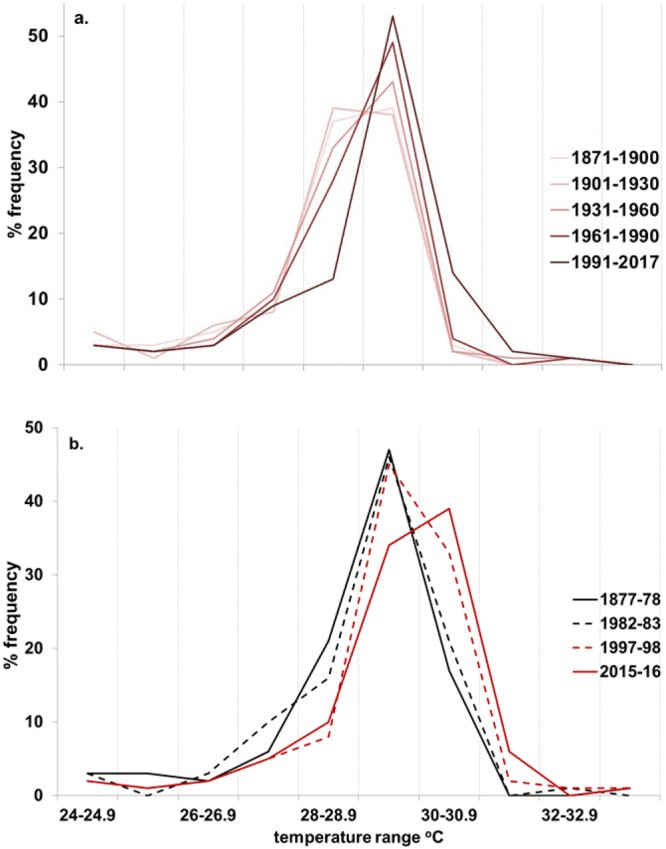
Frequency distribution of annual maximum monthly SST for 100 reef locations for (**a**) 5 sub-periods since the late 19th century and (**b**) 4 strong El Niño events.

The distribution of maximum monthly SST at the 100 locations has also progressively shifted since the late 19^th^ century (Fig. 4a). The maximum monthly SST associated with major El Niño events has similarly changed through time (Fig. 4b). The majority of SST maxima at the 100 reef locations were in the range 29–30 °C during the 1877–1878, 1982–1983 and 1997–1998 events but this has now shifted for 2015–2016 to 30–31 °C.

**Figure 5:**
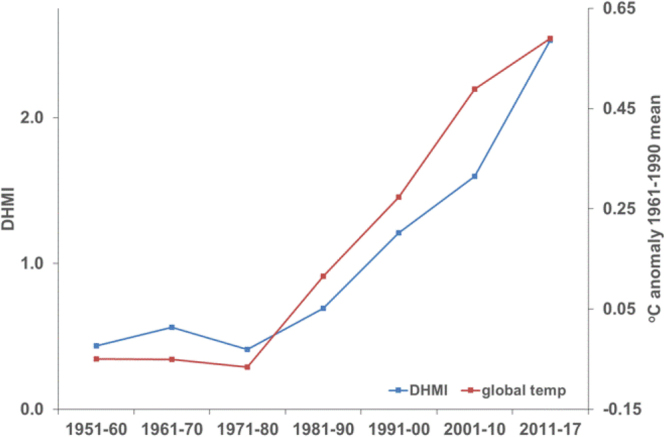
Average degree heating month index DHMI (blue) and global land and sea temperature (red) for 10-year periods, 1951–1960 through 2001–2010 and most recent 8-year period, 2011–2017.

Post 1950, when the SST data are most reliable, there has been a marked increase in thermal stress at the reef sites since the 1980s that closely matches the increase in global land and sea temperatures (Fig. 5). The most recent 8 years, 2011–2017, experienced greater thermal stress than the 2000s with each decade since the 1980s warmer than the preceding one.

Assuming current linear rates of warming continue for the 100 reef locations (Table S1), the estimated level of thermal stress if global warming is constrained to 1.5 °C is 8.5 DHMI, for 2.0 °C is 14.6 DHMI and for 3.0 °C is 30.1 DHMI (Table 1). These estimated levels of thermal stress are ~7, 11 and 23 times, respectively, the level of thermal stress that these reefs experienced in the pre-industrial event of 1878; and ~2, 3 and 6 times the level of thermal stress experienced in 2016. Regionally, the escalation in thermal stress estimated for the different global warming scenarios is greatest for Southeast Asia and least for the Pacific Ocean (Table 1).

## Discussion

Large-scale mass coral bleaching events have increased in frequency, extent and intensity since the latter decades of the 20^th^ century^28,35^. This breakdown of the fundamental symbiosis at the heart of healthy tropical coral reefs already compromises the future of these charismatic, socio-economically valuable and spectacularly diverse ecosystems. Projections of future stress as the world continues to warm, even under low emissions scenarios, place coral reefs at high to very high risk of continued degradation^12,13,36^. Even with the aspirational Paris Agreement target of limiting global warming to 1.5 °C above pre-industrial levels, it is estimated that after 2050, 70% of the world’s reefs will be at risk of severe degradation^37^. Unfortunately, the effects of a warming world is not a future event for coral reefs; it is already happening and compounds ongoing degradation of these dynamic, yet fragile, ecosystems due to overfishing, coastal development and pollution^38^.

This existing vulnerability has been exemplified in the global mass coral bleaching event of 2015–2016^24,27,28^. In this study we focussed on the thermal histories at 100 globally-distributed reef locations. The 2015–2016 bleaching coincided, as have several recent mass coral bleaching events, with a strong El Niño event, when large parts of the tropical ocean experience unusually warm SST^26,39,40^. The increased potential for thermal stress causing bleaching on tropical coral reefs during El Niño events is now compounded by warming of the tropical oceans as the global climate system warms^33^, i.e. baseline SST are now warmer than they were. There is also spatial variation in the magnitude of warming of the tropical oceans^41^ but overall the level of thermal stress that can potentially result in bleaching is clearly increasing^36^. The average global land and sea temperature is commonly used to quantify the rate of past and potential future warming^33^. This metric disguises, however, spatial variations in the rate of warming, with land areas warming faster than the oceans and higher latitudes warming faster than lower latitudes^42^. Thus, 1 °C of global warming does not equate to 1 °C warming of the tropical oceans^41^. SST for 1,670 global tropical coral reef locations have warmed 71% of the global average rate over the period 1880–2017.

For the 100 selected coral reef locations examined in this study, the level of thermal stress in 1983 (1.6 DHMI), the first documented mass coral bleaching^10^, was nearly 25% higher than that experienced during the major 1878 El Niño event^29,30^. We do not know, due to lack of observations, whether any corals bleached during this ‘pre-industrial’ El Niño but based on recent years (e.g. 1988 and 2006) with similar levels of stress as in 1878 (1.3 DHMI; Fig. 2) for which we have observations, it would seem likely that only some localised bleaching (affecting <15% of locations) might have occurred, if any. Thermal stress during the 1997–1998 El Niño, which affected nearly every coral reef region^17,18^, was ~3 times that experienced in the late 19^th^ century. For the two years of the most recent, 2015–2016 event, this increased to 4.9 times the ‘pre-industrial’ level of El Niño-induced stress at the 100 selected locations. This increase in the level of thermal stress to coral reefs during El Niño events has occurred with the relatively modest amount of global warming observed to date (1880–2017) of ~0.92 °C. As the world warms we are also seeing a shift in the distribution of SST on tropical coral reefs^38^ (Fig. 4).

The level of thermal stress (4.7 DHMI) experienced by the 100 coral reef locations in 2016 was unprecedented in the instrumental record, 1871–2017, and exceeded that experienced in 1998 (3.9 DHMI). Given the global extent of coral reefs affected by the 2015–2016 bleaching event^24,28^, a recurrence of thermal stress of similar magnitude in the near future (as is likely with continued global warming) would be of significant consequence for the maintenance of the world’s reefs. Continued warming of the tropical oceans combined with projected more frequent and severe El Niño events^43,44^ will continue to exacerbate the levels of thermal stress experienced by coral reefs^12^. Although there may be regional and local-scale variations in the rates of warming and thermal stress to coral reefs^45,46^, the overall trajectory will be towards higher levels of thermal stress with, as demonstrated here, each strong El Niño event resulting in a higher level of stress than previous ones and the emergence of significant bleaching in non-El Niño years^28^.

There is considerable debate as regards the potential for corals (and the coral-algal holobiont) to acclimatize or adapt to warming tropical oceans and increasing frequency and intensity of mass bleaching events^47,48^. Modelling studies suggest that corals need to increase their thermal tolerance (i.e. temperature at which they bleach) by about 1.5 °C to significantly delay the onset of more frequent bleaching events^49^. As a consequence, more radical interventions to help corals survive a rapidly changing thermal environment (such as assisted evolution, habitat engineering)^50^, concentrating management on naturally resistant/resilient reefs^51^ and a changed approach to reef governance are actively being explored^48^.

Coral reefs are dynamic ecosystems and can, given time, recover from severe stress events, though often with modified community makeup^9^. For example, there was a remarkable recovery of coral cover on the isolated Scott Reef off Western Australia 12 years after it was devastated by bleaching in 1998^52^. Unfortunately, this reef was again severely impacted by mass coral bleaching in 2016^27^. Some reefs have witnessed consecutive years of bleaching, e.g. Hawaiian Archipelago in 2014 and 2015^24^ and Australia’s Great Barrier Reef in 2016 and 2017^53^. The interval between thermal stress events has also shortened with the 0.92 °C of global warming observed to date^28^. Even the aspirational Paris Agreement target of constraining global warming to 1.5 °C above pre-industrial levels is unlikely to be sufficient to prevent drastic modifications and reconfigurations of the community structure and make-up of coral reefs. For the 100 reef locations examined here and given current rates of warming, the 1.5 °C global warming target represents twice the thermal stress they experienced in 2016. The 2 °C global target would result in 3 times the 2016 level of thermal stress and 3 °C, which is currently being tracked with the NDCs^32^, would be over 6 times the 2016 level of stress. The optimistic global targets of +1.5 °C and 2.0 °C, even if achieved, are unlikely to provide the thermal environment necessary for the maintenance of coral reef communities typical of the mid-20^th^ century.

## Data and Methods

Monthly SST were obtained from the HadISST1 data set, January 1871- December 2017^54^ for (a) the 1-degree latitude by longitude boxes containing 100 coral reef locations with robust records of bleaching events, 1980–2016^28^ (Table S1), and 2) 1,670 1-degree latitude by longitude boxes containing tropical coral reefs^41^. It should be noted that, as with all ‘reconstructed’ SST data sets, due to lack of full observational coverage, the HadiSST1 data set is probably most reliable since the 1950s^40^. Global land and sea temperature anomalies from the 1961–1990 baseline average were obtained from the HadCRUT4 data set^55^, 1850–2017. Monthly values of the Niño 3.4 SST index of El Niño-Southern Oscillation (ENSO) were obtained from the HadISST1 data set and the NOAA Climate Prediction Center, 1871–2017.

Rates of warming of annual average global land and sea temperature (calculated from HadCRUT4 data set) and tropical coral reef SST (calculated from HadISST1 data set) were compared over the period 1880–2017 based on linear trend analysis^33^. The ratio of the annual linear trend of tropical SST to global average land and sea temperature (expressed as a percentage) was also mapped throughout the tropical oceans, between 30.5°N-30.5°S, to provide an indication of the spatial variation in warming over the period 1880–2017. For each of the selected 100 coral reef boxes, the level of thermal stress was calculated based on annual degree heating months, 1871–2017, calculated from the HadISST1 data set. This combines both the magnitude and duration of SST anomalies by summing positive monthly anomalies from the 1961–1990 average monthly maximum SST for each year and site. Degree heating month values were totalled over 12 month periods for each location and year for January to December for 72 locations, from December-November at 18 locations, November to October at 8 locations and October to September at 2 locations. These different 12-month periods allowed for Southern Hemisphere locations where seasonal SST maxima can span two calendar years (see Table S1). The degree heating month values at each location were then standardized by the respective, 1961–1990, standard deviation to allow for differences in the variance of the values amongst the 100 reef locations; the median standard deviation of degree heating months for all 100 locations was 0.41° months but some sites, primarily, in the near-equatorial Pacific, have much higher values e.g. 2.07° months for Kiribati and 1.91° months for Ecuador. Standardised values were then averaged to form a Degree Heating Month Index (DHMI) for the 100 reef locations and for four regions: the Caribbean and Atlantic (22 locations), the Indian Ocean and Middle East (28 locations), the Pacific Ocean (36 locations) and Southeast Asia (14 locations) (see Table S1).

The level of thermal stress at the 100 selected locations was also estimated for scenarios where global warming is limited to 1.5 °C, 2.0 °C or 3 °C. These estimates were based on the assumption that the current rate (1880–2017) of SST warming at a particular reef location is maintained into the future. Although this may not necessarily be the case, this simple scaling approach can provide a reasonable indication of what the future may hold^56^. For each location the ratio of monthly warming relative to global land and sea warming (0.92 °C) was calculated based on linear trend analysis, 1880–2017. Taking this ratio and the amount of warming that has already occurred, monthly SST were then calculated at each site for 1.5°, 2.0 °C and 3 °C of global warming. The DHMI was then calculated (as above) as the average standardised value for these three cases.

### Data availability

The HadISST1 data set is available from the UK Meteorological Office Hadley Centre: https://www.metoffice.gov.uk/hadobs/hadisst/index.html; The global land and sea temperatures data set (HadCRUT4) is available from the UK Meteorological Office Hadley Centre: https://www.metoffice.gov.uk/hadobs/hadcrut4/; The Niño 3.4 SST index, 1871–2017, was derived from HadISST1 and the NOAA Climate Prediction Center: http://www.cpc.ncep.noaa.gov/data/indices; Locations and bleaching histories of 100 coral reef locations are available in the Supplementary Material of Hughes *et al*.^31^.

## Electronic supplementary material

Supplementary Material
