# Supplementary material for: Increasing thermal stress for tropical coral reefs: 1871–2017
Source: Sci Rep. 2018 Apr 17;8:6079. doi: 10.1038/s41598-018-24530-9 (PMC5904187; doi:10.1038/s41598-018-24530-9)
Supplement: Supplementary file 1 — Supplementary Material [file 41598_2018_24530_MOESM1_ESM.pdf]

Supplementary Material

Increasing thermal stress for tropical coral reefs: 1871-2017

J.M. Lough, K.D. Anderson and T.P. Hughes

Table S1: 100 reef locations with bleaching histories (1980-2016)[31] by region and central latitude and longitude of nearest 1-degree box for which HadISST1 data were extracted. Also shown is amount of warming, 1880-2017, based on linear trend analysis, of annual average, maximum monthly and minimum monthly SST and the average maximum monthly SST, 1961-1990 (baseline for calculating heating degree month values).

**Site**	**Latitude**	**Longitude**	**Annual average**	**Maximum monthly**	**Minimum monthly**	**Average monthly maximum 1961-90**
**Caribbean & Atlantic**						
Bermuda	32.5ºN	64.5ºW	0.82ºC	0.82ºC	0.74ºC	27.55±0.24ºC
Gulf of Mexico (Texas Flower Garden)	27.5ºN	93.5ºW	0.35ºC	0.25ºC	0.38ºC	29.73±0.36ºC
Bahamas	25.5ºN	77.5ºW	0.41ºC	0.28ºC	0.30ºC	29.28±0.27ºC
Florida Keys	24.5ºN	81.5ºW	0.39ºC	0.49ºC	-0.04ºC	29.75±0.29ºC
Mexico (Yutacan)	22.5ºN	88.5ºW	0.46ºC	0.44ºC	0.26ºC	29.17±0.31ºC
Cuba	20.5ºN	79.5ºW	0.53ºC	0.58ºC	0.46ºC	29.62±0.24ºC
Cayman Islands	19.5ºN	81.5ºW	0.58ºC	0.60ºC	0.55ºC	29.42±0.21ºC
British Virgin Islands	18.5ºN	64.5ºW	0.64ºC	0.71ºC	0.60ºC	28.77±0.34ºC
Jamaica	18.5ºN	77.5ºW	0.59ºC	0.56ºC	0.66ºC	29.37±0.22ºC
Puerto Rico	18.5ºN	67.5ºW	0.64ºC	0.66ºC	0.65ºC	28.87±0.30ºC
Belize	17.5ºN	87.5ºW	0.42ºC	0.34ºC	0.48ºC	29.45±0.38ºC
Dominican Republic	17.5ºN	69.5ºW	0.69ºC	0.74ºC	0.68ºC	28.86±0.29ºC
St Croix & US Virgin Islands	17.5ºN	64.5ºW	0.67ºC	0.78ºC	0.61ºC	28.83±0.34ºC
Honduras	16.5ºN	85.5ºW	0.57ºC	0.46ºC	0.68ºC	29.21±0.30ºC
Barbados	13.5ºN	59.5ºW	0.78ºC	0.88ºC	0.75ºC	28.71±0.26ºC
Bonaire	12.5ºN	68.5ºW	0.90ºC	1.04ºC	0.77ºC	28.65±0.32ºC
Curacao	12.5ºN	69.5ºW	0.92ºC	1.07ºC	0.76ºC	28.62±0.34ºC
Tobago	11.5ºN	60.5ºW	0.86ºC	0.99ºC	0.75ºC	28.75±0.29ºC
Venezuela	11.5ºN	66.5ºW	0.80ºC	0.90ºC	0.67ºC	28.46±0.30ºC
Columbia (Caribbean)	10.5ºN	76.5ºW	0.77ºC	0.84ºC	0.69ºC	28.82±0.28ºC
Costa Rica (Caribbean)	10.5ºN	82.5ºW	0.95ºC	1.10ºC	0.71ºC	28.65±0.27ºC
Panama (Caribbean)	9.5ºN	80.5ºW	0.94ºC	0.96ºC	0.88ºC	28.63±0.32ºC
**Indian Ocean & Middle East**						
Red Sea (Hurghada, Egypt)	27.5ºN	34.5ºE	0.89ºC	1.13ºC	0.65ºC	27.47±0.51ºC
Arabian Gulf (Abu Dhabi, UAE)	24.5ºN	54.5ºE	0.42ºC	-0.26ºC	0.56ºC	32.37±0.40ºC
Red Sea (Thuwal, Saudi Arabia)	22.5ºN	38.5ºE	0.66ºC	0.59ºC	0.65ºC	30.64±0.37ºC
Red Sea (Al Lith, Saudi Arabia)	21.5ºN	38.5ºE	0.66ºC	0.48ºC	0.83ºC	30.91±0.33ºC
Lakshadweep	8.5ºN	73.5ºE	0.86ºC	0.81ºC	0.82ºC	30.05±0.29ºC
Sri Lanka	7.5ºN	79.5ºE	0.85ºC	0.85ºC	0.80ºC	29.76±0.36ºC
Maldives	3.5ºN	73.5ºE	0.89ºC	0.80ºC	0.84ºC	29.49±0.30ºC
Kenya†	3.5ºS	41.5ºE	1.05ºC	1.07ºC	0.88ºC	29.17±0.28ºC
Seychelles	4.5ºS	55.5ºE	1.10ºC	1.08ºC	0.93ºC	29.55±0.31ºC

Chagos Archipelago†	6.5ºS	71.5ºE	0.71ºC	0.55ºC	0.62ºC	29.01±0.27ºC
Tanzania†	7.5ºS	40.5ºE	0.96ºC	1.00ºC	0.75ºC	29.14±0.38ºC
Aldabra (Seychelles) †	9.5ºS	46.5ºE	0.62ºC	0.75ºC	0.41ºC	29.22±0.30ºC
Christmas Island (WA)	10.5ºS	105.5ºE	0.56ºC	0.58ºC	0.50ºC	28.97±0.44ºC
Comores†	11.5ºS	43.5ºE	0.76ºC	0.80ºC	0.65ºC	29.23±0.35ºC
Ashmore Reef (WA) ‡	12.5ºS	123.5ºE	0.40ºC	0.51ºC	0.34ºC	29.99±0.32ºC
Cocos Island	12.5ºS	96.5ºE	0.50ºC	0.49ºC	0.58ºC	28.29±0.37ºC
Mayotte†	12.5ºS	45.5ºE	0.72ºC	0.75ºC	0.71ºC	29.11±0.34ºC
Scott & Seingapatam Reefs (WA) ‡	13.5ºS	121.5ºE	0.36ºC	0.53ºC	0.26ºC	29.85±0.42ºC
Kimberley Coast (WA) ‡	14.5ºS	123.5ºE	0.30ºC	0.31ºC	0.26ºC	29.95±0.38ºC
Rowley Shoals (WA) †	17.5ºS	119.5ºE	0.35ºC	0.34ºC	0.43ºC	29.61±0.36ºC
Madagascar†	19.5ºS	43.5ºE	0.89ºC	1.02ºC	0.80ºC	28.86±0.44ºC
Mozambique	19.5ºS	37.5ºE	1.10ºC	1.07ºC	1.11ºC	28.69±0.42ºC
La Reunion†	21.5ºS	55.5ºE	0.68ºC	0.79ºC	0.68ºC	27.77±0.42ºC
Pilbara Coast (WA)	21.5ºS	114.5ºE	0.57ºC	0.49ºC	0.69ºC	28.42±0.47ºC
Ningaloo Reef (WA)	22.5ºS	113.5ºE	0.72ºC	0.78ºC	0.71ºC	27.12±0.53ºC
South Africa	27.5ºS	33.5ºE	0.94ºC	0.95ºC	0.81ºC	27.02±0.41ºC
SW Western Australia	28.5ºS	113.5ºE	0.90ºC	0.97ºC	1.01ºC	23.69±0.53ºC
Mauritius	30.5ºS	58.5ºE	0.70ºC	0.66ºC	0.78ºC	24.08±0.72ºC
**Pacific Ocean**						
Japan northern	29.5ºN	129.5ºE	1.48ºC	1.12ºC	1.53ºC	28.76±0.41ºC
Ryukyu Islands	26.5ºN	128.5ºE	1.29ºC	1.20ºC	1.29ºC	29.13±0.41ºC
Hawaii (NW islands)	25.5ºN	168.5ºW	0.63ºC	0.52ºC	0.73ºC	27.18±0.37ºC
Taiwan	23.5ºN	122.5ºE	2.12ºC	1.68ºC	2.55ºC	29.04±0.40ºC
Hawaii (main islands)	19.5ºN	156.5ºW	0.55ºC	0.63ºC	0.47ºC	27.11±0.46ºC
Johnston Atoll	16.5ºN	170.5ºW	0.17ºC	0.27ºC	0.07ºC	28.24±0.41ºC
CNMI	15.5ºN	145.5ºE	0.52ºC	0.71ºC	0.39ºC	29.43±0.23ºC
Guam	13.5ºN	144.5ºE	0.53ºC	0.77ºC	0.41ºC	29.40±0.30ºC
Republic Marshall Islands	11.5ºN	166.5ºE	0.19ºC	0.18ºC	0.17ºC	29.26±0.36ºC
Costa Rica (Pacific coast)	9.5ºN	84.5ºW	0.84ºC	0.84ºC	0.70ºC	29.06±0.49ºC
Panama (Gulf of Panama)	8.5ºN	79.5ºW	0.88ºC	0.92ºC	0.79ºC	28.53±0.36ºC
Palau	7.5ºN	134.5ºE	0.64ºC	0.79ºC	0.37ºC	29.32±0.32ºC
Panama (Gulf of Chiriqui)	7.5ºN	81.5ºW	0.91ºC	1.12ºC	0.60ºC	28.87±0.39ºC
Columbia (Pacific)	6.5ºN	78.5ºW	0.73ºC	0.86ºC	0.50ºC	28.06±0.47ºC
Kiribati (Christmas Island)	1.5ºN	157.5ºW	0.15ºC	0.24ºC	-0.16ºC	28.24±0.79ºC
Galapagos‡	0.5ºS	89.5ºW	-0.07ºC	0.27ºC	-0.25ºC	26.77±0.86ºC
Ecuador (mainland) ‡	1.5ºS	82.5ºW	-0.03ºC	0.51ºC	-0.21ºC	26.32±0.94ºC
Gilbert Islands (Kiribati)	3.5ºS	172.5ºE	0.52ºC	0.42ºC	0.44ºC	29.68±0.38ºC
Solomon Islands #	8.5ºS	158.5ºE	0.56ºC	0.55ºC	0.58ºC	29.71±0.29ºC

Torres Strait‡	10.5°S	142.5°E	0.56°C	0.65°C	0.51°C	29.36±0.33°C
Northern GBR†	11.5°S	144.5°E	0.71°C	0.68°C	0.82°C	29.33±0.42°C
Samoa‡	13.5°S	172.5°W	0.44°C	0.49°C	0.40°C	29.52±0.35°C
American Samoa†	14.5°S	170.5°W	0.39°C	0.33°C	0.38°C	29.32±0.35°C
Vanuatu†	15.5°S	167.5°E	0.57°C	0.76°C	0.39°C	28.98±0.33°C
Coral Sea (north) †	16.5°S	149.5°E	1.06°C	1.00°C	1.24°C	28.89±0.42°C
Fiji†	16.5°S	177.5°E	0.55°C	0.65°C	0.49°C	29.07±0.37°C
French Polynesia (Society Islands)†	16.5°S	151.5°W	0.35°C	0.34°C	0.31°C	28.91±0.37°C
Central GBR†	19.5°S	148.5°E	0.85°C	0.67°C	1.10°C	28.70±0.40°C
Coral Sea (south)†	20.5°S	154.5°E	0.94°C	1.00°C	1.03°C	28.12±0.42°C
Cook Islands	21.5°S	159.5°W	0.44°C	0.47°C	0.40°C	27.59±0.49°C
New Caledonia	21.5°S	164.5°E	0.67°C	0.72°C	0.77°C	27.38±0.49°C
Southern GBR†	23.5°S	151.5°E	0.86°C	0.70°C	1.04°C	27.69±0.40°C
Moreton Bay (Qld)	27.5°S	153.5°E	1.31°C	1.22°C	1.67°C	26.55±0.47°C
Solitary Islands (NSW)	30.5°S	153.5°E	1.52°C	1.48°C	1.83°C	25.61±0.52°C
South West Rocks (NSW)	30.5°S	153.5°E	1.52°C	1.48°C	1.83°C	25.61±0.52°C
Lord Howe Island (NSW)	31.5°S	158.5°E	0.84°C	0.86°C	1.08°C	24.67±0.59°C
**SE Asia**						
Philippines	14.5°N	119.5°E	0.71°C	0.69°C	0.59°C	29.87±0.34°C
Thailand	10.5°N	101.5°E	0.50°C	0.59°C	0.15°C	30.22±0.33°C
South Vietnam (Con Dao)	8.5°N	106.5°E	0.61°C	0.74°C	0.17°C	29.90±0.43°C
Indonesia (Aceh)	5.5°N	96.5°E	0.91°C	0.87°C	0.83°C	29.65±0.28°C
Indonesia (N Sulawesi/Manado)	1.5°N	123.5°E	0.79°C	0.81°C	0.67°C	29.35±0.26°C
Singapore/Indonesia (Riau)	1.5°N	102.5°E	0.67°C	0.72°C	0.51°C	29.96±0.26°C
Indonesia (central Sulawesi)	0.5°S	120.5°E	0.95°C	1.02°C	0.87°C	29.52±0.18°C
Indonesia (W/S Sumatra)	2.5°S	99.5°E	0.61°C	0.67°C	0.73°C	29.62±0.28°C
Indonesia (Kalimantan)	3.5°S	110.5°E	0.61°C	0.71°C	0.54°C	29.50±0.34°C
Indonesia (S Sulawesi)	4.5°S	118.5°E	0.72°C	0.69°C	0.71°C	29.48±0.36°C
Papua New Guinea (Kimbe Bay) #	4.5°S	150.5°E	0.54°C	0.63°C	0.32°C	29.71±0.32°C
Indonesia (S-E Sulawesi/Wakatobi)	5.5°S	125.5°E	0.58°C	0.66°C	0.44°C	29.39±0.29°C
Indonesia (Java)	6.5°S	109.5°E	0.47°C	0.53°C	0.37°C	29.40±0.38°C
Indonesia (Bali/Lombok) ‡	8.5°S	115.5°E	0.52°C	0.50°C	0.56°C	29.19±0.39°C

† indicates sites where degree months were summed from December to November

‡ indicates sites where degree months were summed from November to October

# indicates sites where degree months were summed from October to September

For all other sites degree months were summed from January to December
